# A multiplex tissue resource for high‐resolution spatial protein profiling in the Human Protein Atlas

**DOI:** 10.1002/pro.70764

**Published:** 2026-08-19

**Authors:** Borbala Katona, Rutger Schutten, Filippa Bertilsson, Feria Hikmet, Per Adelsköld, Mattias Forsberg, Kalle von Feilitzen, Loren Méar, Mathias Uhlén, Cecilia Lindskog

**Affiliations:** ^1^ Department of Immunology, Genetics and Pathology, Cancer Precision Medicine Research Unit Uppsala University Uppsala Sweden; ^2^ Department of Organismal Biology, Nordfertil Research Lab Uppsala, Physiology and Environmental Toxicology Uppsala University Uppsala Sweden; ^3^ Science for Life Laboratory, School of Engineering Sciences in Chemistry, Biotechnology and Health KTH Royal Institute of Technology Stockholm Sweden; ^4^ Department of Women's and Children's Health, Division for Neonatology, Obstetrics, Gynaecology and Reproductive Health Karolinska Institute and Karolinska University Hospital Stockholm Sweden; ^5^ Department of Neuroscience Karolinska Institutet Stockholm Sweden

**Keywords:** antibody‐based proteomics, cell type‐specific expression, functional protein annotation, Human Protein Atlas, human tissues, multiplex immunohistochemistry, protein localization, spatial proteomics, subcellular localization, tissue profiling

## Abstract

Spatially resolved protein expression is essential for understanding tissue organization, cellular specialization, and protein function. The open‐access Human Protein Atlas database (www.proteinatlas.org) has generated an extensive antibody‐based tissue resource for a majority of the human protein‐coding genes using conventional immunohistochemistry, enabling body‐wide annotation of protein expression across normal human tissues and major cell types. However, single‐marker staining often lacks the cellular and subcellular context required to resolve rare cell populations, closely related cell states, or proteins with limited functional characterization. To address this, we established a multiplex tissue resource within the Human Protein Atlas based on a large‐scale multiplex immunohistochemistry workflow. The iterative workflow combines optimized antibody panels targeting established markers of cell identity, tissue organization, cellular state, and subcellular structure with candidate proteins of interest. This allows protein expression to be interpreted directly within intact tissue architecture based on expression overlap between candidate proteins and panel markers. In version 25 of the Human Protein Atlas, 1106 proteins have been analyzed using eight multiplex antibody panels across nine tissue settings, including testis, motile ciliated epithelia, salivary gland, endocrine pancreas, and kidney. These panels resolve biological contexts such as stages of spermatogenesis, Sertoli cell and ciliary subcellular compartments, salivary gland acinar and ductal structures, pancreatic endocrine cell types, and nephron segments. Here, we present the design and implementation of the multiplex tissue resource and demonstrate its utility for refining spatial protein annotation across diverse human tissue systems. By providing high‐resolution spatial context for protein expression in human tissues, this publicly available resource strengthens functional protein annotation and offers a framework for generating new hypotheses about protein roles in normal tissue biology.

## 1 INTRODUCTION

The Human Protein Atlas (HPA, www.proteinatlas.org) was launched in 2003 with the goal of systematically mapping the spatial distribution of human proteins across tissues, organs, and cell types using antibody‐based proteomics (Uhlen et al., 2010; Uhlén et al., 2005). Over the past two decades, the HPA has developed into one of the world's largest biological databases, providing a comprehensive open‐access resource integrating transcriptomics, proteomics, and functional data to characterize protein expression in normal tissues and cell types, cancer tissues, cultured cells, and blood (Álvez et al., 2025; Karlsson et al., 2021; Sjöstedt et al., 2020; Thul et al., 2017; Uhlen et al., 2017; Uhlen et al., 2019; Uhlén et al., 2015). Central to the original HPA effort is the large‐scale application of classical immunohistochemistry (IHC), in which validated antibodies are used to visualize protein expression in formalin‐fixed paraffin‐embedded (FFPE) tissue sections across major human organs. This approach established the first atlas‐scale framework for studying protein localization directly in human tissues and demonstrated the importance of spatially resolved protein analysis for understanding tissue organization, cellular specialization, and disease pathology (Uhlen et al., 2017; Uhlén et al., 2015).

Classical chromogenic IHC remains a cornerstone of pathology and biomarker research because of its robustness, relatively low cost, compatibility with standard clinical workflows, and preservation of tissue morphology (Magaki et al., 2019). The method is technically straightforward, scalable, and widely accessible, making it particularly valuable for routine diagnostics and large‐scale tissue profiling efforts such as the HPA. In addition, chromogenic staining enables direct visualization of protein expression within a spatially intact histological context, allowing cellular localization to be interpreted together with tissue architecture. However, IHC typically only visualizes one protein per tissue section and has limited ability to quantitatively compare protein abundance across cell types or characterize complex cellular states. Interpretation frequently relies on morphological assessment by the human eye, which can make it difficult to accurately identify rare or phenotypically similar cell populations based on histology alone. This challenge becomes particularly pronounced for proteins with limited prior characterization, where the expressing cell type or subcellular localization may not be known in advance. In such cases, single‐marker IHC often provides insufficient contextual information to confidently assign expression patterns to specific cell types or cellular states.

The need to improve functional annotation of poorly characterized proteins is also a central goal of the Human Proteome Project (HPP), coordinated by the Human Proteome Organization (HUPO) (Adhikari et al., 2020). The HPP Grand Challenge, “A Function for Every Protein,” aims to advance functional understanding of the human proteome and includes a Function Evidence (FE) scoring system that ranks the current level of evidence for protein function (Omenn et al., 2024). In this system, proteins with strong experimental support for function are assigned higher‐confidence functional evidence, whereas proteins with limited or unknown functional information are highlighted as priorities for further studies. While spatial localization alone is not sufficient to determine protein function, it can provide an important first clue by placing a protein in a defined biological context. This is particularly relevant for tissue enriched or cell type enriched proteins, where localization to a specific tissue structure, cell type, differentiation stage, or subcellular compartment can guide more focused functional studies.

The standard Tissue Resource dataset in the HPA is based on IHC and has primarily focused on the major cell types within each tissue, encompassing 78 annotated cell types across 44 major normal human tissues. In parallel, targeted efforts have been undertaken to increase cellular resolution in selected organs. For example, a dedicated study of human testis expanded the previous annotation scheme from two broad cell populations to eight distinct germ cell and somatic cell categories through close collaboration with experts in reproductive biology (Pineau et al., 2019). However, subsequent automated image analysis demonstrated that even domain experts may face challenges in consistently interpreting complex or ambiguous staining patterns, particularly for proteins with restricted or previously unknown expression profiles (Ghoshal et al., 2021). These observations highlight the limitations of morphology‐based single‐marker interpretation and underscore the need for multiplexed and computational approaches for robust characterization of cell type and cell state‐specific protein expression patterns.

Recent advances in single‐cell and spatial biology have dramatically transformed the understanding of tissue organization and cellular heterogeneity. In particular, single‐cell RNA sequencing (scRNA‐seq) has enabled systematic classification of diverse cell types and cellular states across human tissues. Nevertheless, scRNA‐seq relies on transcript abundance as a proxy for cellular phenotype, lacks native tissue context, and does not directly measure functional protein levels. This distinction is important because mRNA abundance is often only partially correlated with protein expression due to differences in RNA regulation, protein synthesis, stability, and localization within the cell. Spatial transcriptomics methods have partly addressed the loss of tissue context by enabling in situ mapping of RNA molecules, yet these approaches remain restricted to transcript‐level measurements and require high costs and computational load. At the same time, most studies still rely on a single methodological approach and therefore capture only limited aspects of tissue organization or function.

To increase cellular resolution while preserving spatial tissue context, the HPA established a multiplex tissue resource based on multiplex immunohistochemistry (mIHC) and computational image analysis. This effort builds on the extensive foundation of the HPA Tissue Resource, including validated antibodies, standardized staining protocols, annotated tissue material, and expert‐curated protein expression data across normal human tissues. These prior data enabled selection of antibodies with documented specificity, robustness, tissue distribution, and subcellular localization, while providing a reference framework for designing biologically informative panels and interpreting multiplex staining patterns. By combining established reference markers with candidate proteins of interest, the panels allow protein localization to be assessed in relation to defined tissue structures and cellular contexts. In this way, mIHC provides a protein‐level complement to transcript‐based approaches and supports the generation of functional hypotheses directly from spatial protein expression patterns.

The current multiplex tissue resource encompasses eight antibody panels toward nine different tissues, representing several distinct tissue and cellular systems. In version 25 of the HPA database, 1106 proteins have been analyzed using multiplex tissue profiling, including panels focused on spermatogenesis and Sertoli cell biology in testis, molecular composition of ciliated cells, secretory organization in salivary gland, endocrine cell populations in pancreas, and functional regions of the nephron in kidney. Together, these datasets illustrate the breadth of biological questions that can be addressed through multiplex spatial proteomics, ranging from developmental transitions and cellular differentiation to tissue‐specific functional specialization. In the following sections, the multiplex tissue profiling workflow together with the organ and tissue systems currently analyzed by mIHC within the HPA Tissue Resource will be described in greater detail, with emphasis on the biological insights gained from spatially resolved protein expression analysis.

## 2 BUILDING A MULTIPLEX TISSUE RESOURCE

**FIGURE 1:**
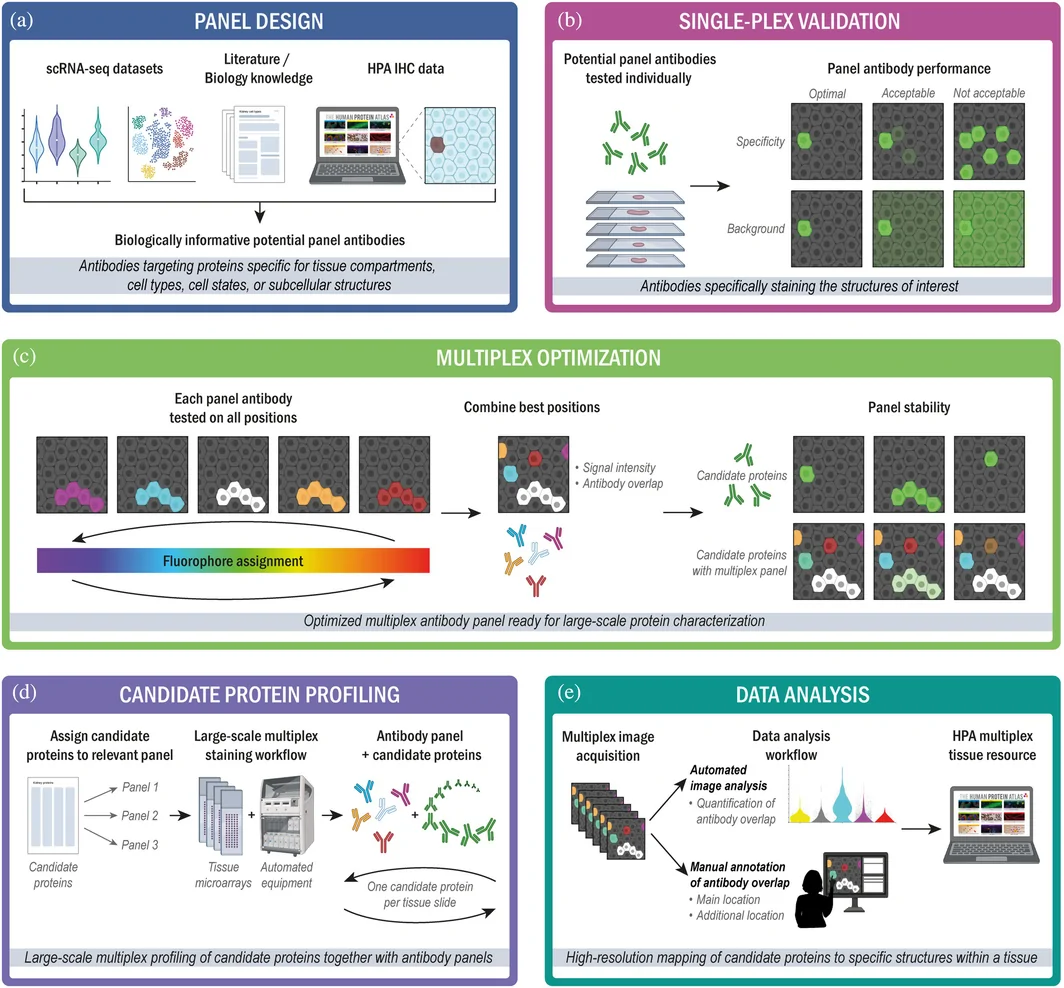
Workflow for multiplex tissue profiling in the Human Protein Atlas (HPA). (a) Design of biologically informative antibody panels by integrating scRNA‐seq data, literature‐based biological knowledge, and prior HPA immunohistochemistry (IHC) data. (b) Selected potential panel antibodies are validated individually using single‐plex immunofluorescence staining on relevant tissue samples to determine compatibility with the multiplex workflow. Only antibodies meeting the quality criteria are advanced to multiplex panel optimization. (c) Multiplex panels are first optimized by testing each antibody on each position in the multiplex workflow, assigning fluorophores and staining order of the antibodies to reflect the best performance. This means that for each specific cell type to be analyzed, all fluorophores are tested, illustrated by different colors. The panel antibodies are then combined into a multiplex setting to validate antibody dilution and determine potential overlap between signals. Finally, a smaller number of candidate proteins are tested together with the optimized antibody panel to ensure stability when adding additional antibodies. Candidate proteins are visualized in green. If the panel antibodies do not pass the multiplex optimization, they will be replaced by new potential panel antibodies, selected as described in (a). (d) Optimized antibody panels are used for large‐scale multiplex profiling, assigning each candidate protein to the most biologically relevant panel. The usage of tissue microarrays and automated staining equipment aids in setting up a scalable workflow. In each experiment, one candidate protein is combined with an antibody panel in the same tissue section. (e) Multiplex images are acquired and analyzed by both automated image analysis and manual annotation to assess antibody overlap, main and additional locations, and quantitative spatial expression patterns. The resulting high‐resolution images and protein localization data are incorporated into the HPA multiplex tissue resource.

A central component of the multiplex tissue profiling workflow is the design of biologically informative antibody panels capable of resolving cellular heterogeneity and tissue organization within complex human tissues (Figure 1a). Because the number of proteins that can be simultaneously visualized remains limited compared with transcriptome‐wide approaches, careful marker selection is essential to maximize biological interpretability and cellular resolution. The overall goal of panel design is therefore not only to identify major cell types but also to capture differentiation states, spatial organization, and functionally specialized cellular populations.

Panel development within the HPA multiplex tissue resource is based on the integration of several complementary sources of information. In‐depth analysis of scRNA‐seq datasets, including the data integrated in the Single Cell Resource, provides an important starting point for identifying transcriptionally distinct cell populations and candidate marker genes with cell type or cell state‐specific expression patterns. The scRNA‐seq datasets are complemented with prior biological knowledge from the literature, together with the extensive single‐marker IHC data already available within the HPA Tissue Resource. Capturing specific cell states or cellular functions is particularly important in tissues characterized by continuous differentiation processes or pronounced cellular heterogeneity, where closely related structures may be difficult to distinguish morphologically. Particular emphasis is placed on selecting panel markers with complementary rather than highly overlapping expression patterns to maximize biological resolution within each panel. When applicable, the staining patterns of all panel markers used in the HPA multiplex tissue resource correlate with mRNA levels based on scRNA‐seq.

Before incorporation into multiplex workflows, antibodies are initially evaluated in a single‐plex format to confirm preservation of staining patterns observed in chromogenic IHC together with sufficient signal intensity and specificity under immunofluorescence conditions (Figure 1b). Antibodies showing inconsistent staining, excessive background, or reduced compatibility with the immunofluorescence setting are replaced by alternative antibodies targeting the same protein or by antibodies targeting other markers with similar biological specificity. Fluorophore assignments are adjusted to balance signal intensity across markers with different expression levels. Low‐abundance proteins are preferentially paired with brighter fluorophores, whereas highly expressed proteins are assigned to dimmer channels to improve overall panel balance and image quality. In addition, the sequential order of antibodies within the multiplex workflow represents an important optimization parameter, as repeated heating and stripping cycles may differentially affect epitope stability and antibody performance. Consequently, some antibodies retain specificity only at particular staining positions within the sequence, requiring iterative evaluation of antibody order to preserve staining quality and reproducibility across the full panel (Figure 1c).

Once the core multiplex panels have been established and optimized, they can subsequently be used in a large‐scale framework for characterization of additional candidate proteins. Selection of candidate proteins for mIHC profiling is similar to the selection of panel markers, guided by differential expression analyses, prior biological knowledge, and evaluation of existing chromogenic IHC data within the HPA. Candidate proteins are assigned to a specific antibody panel representing the structures in which their expression is expected to occur and are subsequently stained together with the panel markers within the same tissue section (Figure 1d). Following image acquisition and spectral unmixing, both manual and computational image analysis are used to evaluate co‐localization patterns, tissue distribution, and cell type‐specific expression (Figure 1e). Through this large‐scale and quantitative workflow, multiplex tissue profiling enables systematic high‐resolution characterization of protein expression across detailed tissue structures, thereby supporting functional interpretation beyond what can typically be achieved using conventional IHC or transcriptomics alone.

Multiplex staining for all antibody panels in the HPA multiplex tissue resource was performed on tissue microarrays (TMAs), using duplicate 1 mm cores from a minimum of three different donors per tissue type. All tissue samples were derived from adult individuals, and where applicable, samples from both sexes were included. Only histologically normal tissue samples were used. The cohort was selected to represent normal adult tissue morphology suitable for antibody‐based spatial protein profiling, rather than to assess donor‐related variation.

To demonstrate how the multiplex workflow is applied across different biological contexts, the following sections describe the antibody panels currently included in the HPA multiplex tissue resource, highlighting representative examples of candidate protein localization. Together, these examples illustrate how multiplex antibody panels can support a more detailed functional interpretation of protein expression than can typically be achieved using single‐marker IHC alone.

## 3 SPERMATOGENESIS AND SERTOLI CELL BIOLOGY IN TESTIS

The testis is characterized by a highly ordered differentiation process in which germ cells progress from spermatogonial stem and progenitor cells to mature spermatozoa within the seminiferous tubules. This process, known as spermatogenesis, includes mitotic proliferation, meiotic division, and extensive post‐meiotic remodeling, and is supported intratubularly by Sertoli cells that provide the structural and regulatory environment required for germ cell development. Because germ cells pass through closely related transitional states and are intimately associated with Sertoli cells, assigning protein expression to specific developmental stages or somatic compartments can be challenging using morphology or single‐marker IHC alone.

**FIGURE 2:**
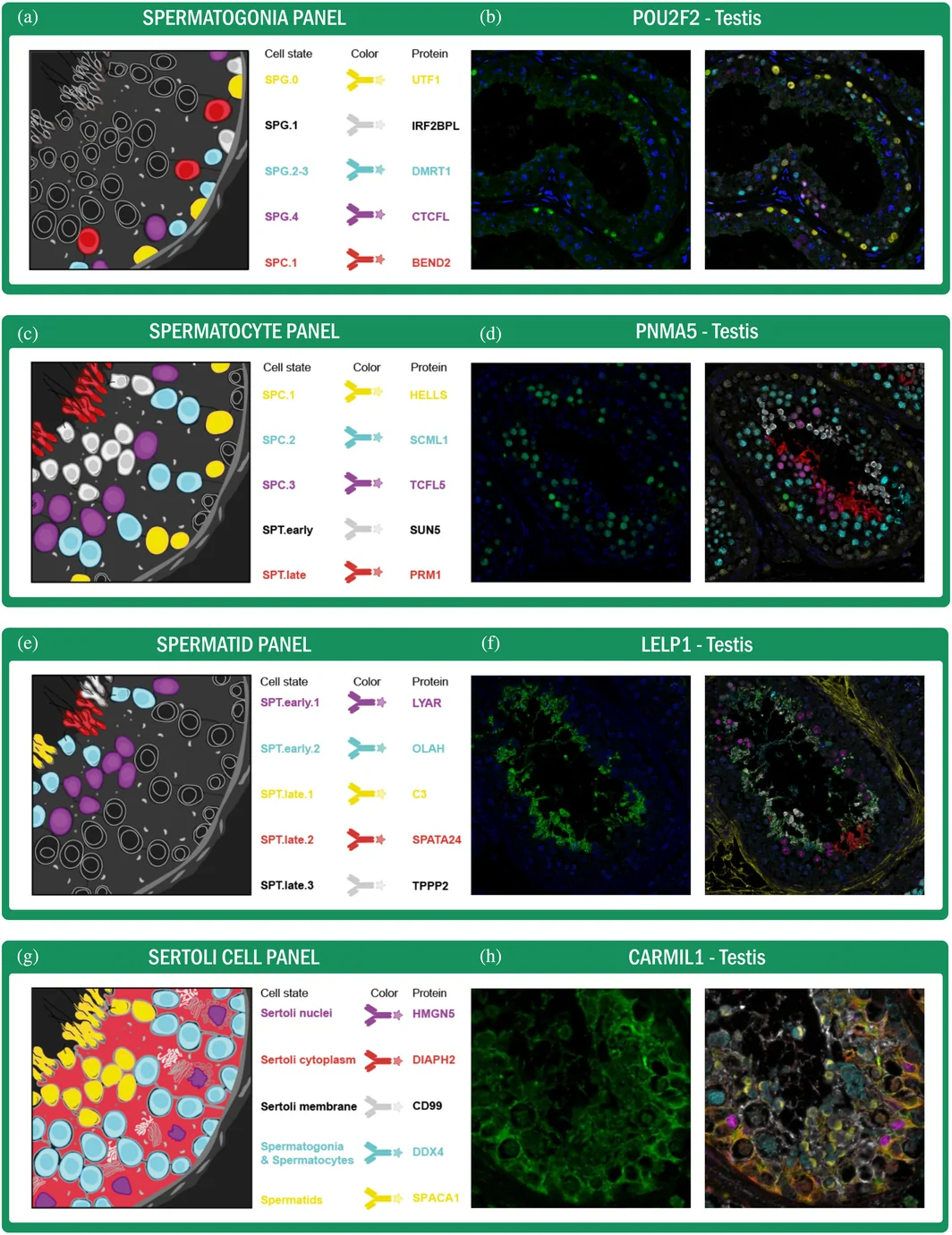
Multiplex tissue profiling of selected candidate proteins across spermatogenesis and Sertoli cell compartments. (a) Overview of the spermatogonia antibody panel, resolving early germ cell states from spermatogonial stem/progenitor cells to preleptotene spermatocytes. (b) Multiplex staining of POU2F2 in testis (left) shows localization to the SPG.0 cell state, as seen by overlap with UTF1 (right). (c) Overview of the spermatocyte antibody panel, distinguishing defined stages of spermatocyte differentiation and meiotic progression. (d) PNMA5 shows nuclear localization in spermatocytes (left), based on signal overlap with SCLM1assigned to the SPC.2 cell state (right). (e) Overview of the spermatid antibody panel, resolving early and late spermatid states during post‐meiotic maturation. (f) LELP1 shows localization to late spermatids (left), with signal assigned to the SPT.late.3 cell state based on overlap with TPPP2. (g) Overview of the Sertoli cell antibody panel, distinguishing Sertoli cell nuclei, cytoplasm, and membrane‐associated compartments together with adjacent germ cell populations. (h) CARMIL1 (left) is determined to localize to the Sertoli cell cytoplasm, based on overlap with DIAPH2.

To capture this complexity, the HPA multiplex tissue resource includes four testis antibody panels focused on spermatogonia, spermatocytes, spermatids, and Sertoli cells (Table 1 and Figure 2). The spermatogonia panel follows early germ cell differentiation toward meiotic entry; the spermatocyte panel captures meiotic progression, and the spermatid panel resolves post‐meiotic maturation during spermiogenesis. The Sertoli cell panel complements these germ cell panels by enabling proteins to be interpreted in relation to Sertoli cell cytoplasm, membrane‐associated regions, and nuclei.

**TABLE 1:** Overview of the antibody panels in the HPA multiplex tissue resource.

Panel	Biological question	Cell type/state/compartment	Marker protein
Spermatogonia (testis)	To resolve early germ cell differentiation and mitotic expansion from spermatogonial stem/progenitor states toward meiotic entry.	Spermatogonia 0	UTF1
		Spermatogonia 1	IRF2BPL
		Spermatogonia 2–3	DMRT1
		Spermatogonia 4	CTCFL
		Spermatocytes 1	BEND2
Spermatocytes (testis)	To map protein expression across meiotic progression and identify proteins associated with distinct spermatocyte stages.	Spermatocytes 1	HELLS
		Spermatocytes 2	SCML1
		Spermatocytes 3	TCFL5
		Spermatids early	SUN5
		Spermatids late	PRM1
Spermatids (testis)	To characterize protein expression during post‐meiotic maturation and structural remodeling in spermiogenesis.	Spermatids early 1	LYAR
		Spermatids early 2	OLAH
		Spermatids late 1	C3
		Spermatids late 2	SPATA24
		Spermatids late 3	TPPP2
Sertoli cells (testis)	To assign protein expression to Sertoli cell compartments and distinguish Sertoli‐associated signals from adjacent germ cell populations.	Sertoli nuclei	HMGN5
		Sertoli cytoplasm	DIAPH2
		Sertoli membrane	CD99
		Spermatogonia and spermatocytes	DDX4
		Spermatids	SPACA1
Ciliated cells	To map candidate proteins to defined compartments of motile ciliated cells across respiratory and female reproductive tissues.	Nucleus	FOXJ1
		Cytoplasm	AGR3
		Transition zone	NPHP4
		Rootlet	CROCC
		Cilium	DNAH9
Salivary gland	To resolve protein expression across secretory acinar cells, ductal compartments, and specialized ion‐transporting epithelial cells.	Small ducts	SLC13A2
		Serous acini	LPO
		Ionocytes	FOXI1
		Large ducts	ATP6V1B1
		Mucus acini	MUC5B
Pancreas (endocrine)	To assign protein expression to distinct endocrine cell populations within the islets of Langerhans.	Beta cells	INS
		Alpha cells	GCG
		Delta cells	SST
		PP‐cells	PPY
		Epsilon cells	GHRL
Kidney	To localize candidate proteins to major nephron and glomerular compartments involved in filtration, transport, and urine concentration.	Endothelial cells	CD34
		Collecting ducts	AQP2
		Podocytes	PTPRO
		Distal tubules (subset)	CASR
		Proximal tubules	ACSM2A/B

Using these four antibody panels, the HPA Tissue Resource has analyzed 700 proteins in testis, including 235 proteins with the spermatogonia panel, 276 proteins with the spermatocyte panel, 89 proteins with the spermatid panel, and 100 proteins with the Sertoli cell panel (Table S1). Mapping candidate proteins to specific stages and compartments of the seminiferous epithelium is important for understanding normal fertility and the molecular regulation of human spermatogenesis. The dataset also enables comparison between RNA expression patterns derived from scRNA‐seq and protein localization observed by mIHC, providing an opportunity to explore RNA‐to‐protein concordance and identify cases where transcript abundance does not directly reflect protein expression or localization (Hikmet et al., 2024). In this way, the testis panels can support functional interpretation of less‐characterized proteins and generate hypotheses about their potential roles in male fertility.

An example of a protein characterized by using the spermatogonia panel is POU2F2 (FE3), a lymphoid‐cell specific transcription factor important for immunoglobulin production (Scheidereit et al., 1988; Strubin et al., 1995). In some cancers, POU2F2 has been associated with tumor‐promoting properties, including increased cell proliferation (Luo et al., 2021; Yang et al., 2021). Our results show POU2F2 localization in Spermatogonia 0 cell state with no detectable overlap in the other spermatogonia cell states (Figure 2a,b).

PNMA5 (FE4) is one of the proteins analyzed by the spermatocyte panel. It is associated with increased cell proliferation, invasion, and migration in colorectal, prostate, and ovarian cancer cells (Lin et al., 2022; Qiao et al., 2020; Zhi et al., 2025), and suggested to play an important role in meiosis based on mouse oogenesis models (Zhang et al., 2017). In our analysis, PNMA5 showed nuclear staining in Spermatocytes 2, which could indicate involvement in meiosis also during human spermatogenesis (Figure 2c,d).

The spermatid panel examined proteins such as LELP1 (FE5), among others, which may be involved in the formation of the cornified envelope, that is, keratinocyte cell membrane. Polymorphism of the LELP1 gene and mRNA level changes are associated with atopic dermatitis and idiopathic nonobstructive azoospermia (Chen et al., 2023; Trzeciak et al., 2016; Trzeciak et al., 2017). We showed LELP1 protein localization exclusively in the Spermatids late 3 cell state (Figure 2e,f).

One of the proteins examined by using the Sertoli cell panel is CARMIL1 (FE2), a cytoskeleton and cell membrane associated protein. It plays a key role in actin assembly and cell migration (Liang et al., 2009). CARMIL1 is localized to the cytoplasm of Sertoli cells according to our mIHC data (Figure 2g,h).

## 4 THE MOLECULAR COMPOSITION OF CILIATED CELLS

Motile cilia are specialized microtubule‐based organelles that extend from the surface of epithelial cells and generate coordinated movement of fluid across tissue surfaces. They are found in several tissues, including the respiratory tract and female reproductive tract, where they contribute to processes such as mucociliary clearance and movement of gametes and fluids (Li & Winuthayanon, 2017; Wallmeier et al., 2020). Defects in motile cilia formation or function are associated with a group of diseases known as ciliopathies, including primary ciliary dyskinesia, highlighting the importance of identifying proteins involved in ciliary structure and motility (Newman et al., 2023; Wallmeier et al., 2020).

**FIGURE 3:**
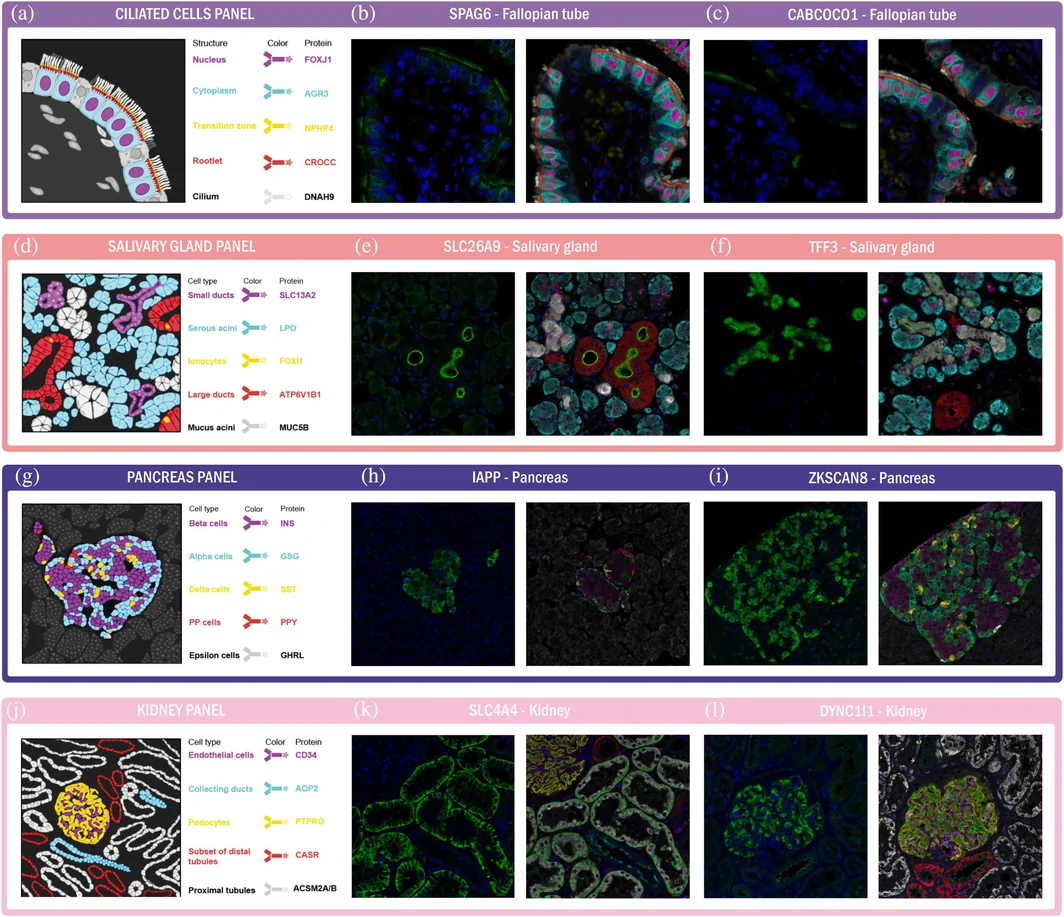
Multiplex tissue profiling of selected candidate proteins in ciliated cells, salivary gland, pancreas, and kidney. (a) Overview of the ciliated cell antibody panel used to define subcellular compartments, including nucleus, cytoplasm, transition zone, rootlet, and cilium. (b) Multiplex staining of SPAG6 in fallopian tube shows localization to the cytoplasm and transition zone, whereas CABCOCO1 (c) is localized to the ciliary compartment. (d) Overview of the salivary gland antibody panel, resolving small ducts, serous acini, ionocytes, large ducts, and mucous acini. (e) SLC26A9 localizes to large ducts, whereas TFF3 (f) is detected in mucous acini. (g) Overview of the endocrine pancreas antibody panel, distinguishing beta, alpha, delta, PP, and epsilon cells within the islets of Langerhans. (h) IAPP localizes to beta cells, whereas ZKSCAN8 (i) shows selective localization to alpha cells. (j) Overview of the kidney antibody panel, resolving endothelial cells, collecting ducts, podocytes, a subset of distal tubules, and proximal tubules. (k) SLC4A4 localizes to proximal tubules, whereas DYNC1I1 (l) is detected in glomeruli, with signal assigned to podocytes.

To characterize motile ciliated cells in the HPA multiplex tissue resource, a cilia antibody panel was designed to combine markers of ciliated cell identity with proteins representing distinct ciliary compartments and functions (Bertilsson et al., 2026) (Table 1). Together, these markers provide a spatial and functional framework for interpreting candidate protein expression in relation to motile ciliated cells and their subcellular organization, including the cytoplasm, nucleus, rootlet, transition zone, and cilium (Figure 3a).

Using the cilia panel, 200 proteins have been analyzed in version 25 of the HPA Tissue Resource across five tissues containing motile ciliated cells: bronchus, nasopharynx, cervix, endometrium, and fallopian tube (Table S1). Analysis of both shared and tissue‐specific patterns of motile cilia‐related proteins contributes to a deeper understanding of the coordinated organization of proteins involved in cilia assembly, structure, and beating. By placing candidate proteins within defined tissue‐specific and subcellular contexts of ciliated cells, the dataset can reveal markers of ciliated epithelial specialization and support studies of molecular mechanisms underlying cilia‐related function and disease.

Examples of analyzed proteins include SPAG6 (FE2), a component of motile cilia and sperm flagella essential for their normal structure and function, and CABCOCO1 (FE5), a poorly characterized calcium‐binding protein (Neilson et al., 1999). The loss of SPAG6 has been associated with male infertility, hydrocephalus, pneumonia, and vestibular dysfunction in mice (Li et al., 2021; Sapiro et al., 2002). Our results show that SPAG6 is distributed in both the cytoplasm and the transition zone of ciliated cells across human respiratory tract and fallopian tube, while its location is restricted to the transition zone in endometrium and cervix (Figure 3b).

A few animal studies have detected CABCOCO1 in sperm flagella and found that its inactivation led to flagella malformations (Kawashima et al., 2016; Wang et al., 2023). Using mIHC, we were able to describe it for the first time in human ciliated cells, where it is specifically located to the cilium compartment in all examined tissues (Figure 3c).

## 5 SECRETORY ORGANIZATION IN SALIVARY GLAND

The salivary glands are highly specialized secretory organs that produce and modify saliva, thereby contributing to oral lubrication, digestion, mucosal protection, antimicrobial defense, and maintenance of the oral environment. Their function depends on the coordinated activity of secretory acinar cells and ductal epithelial cells. Serous and mucous acini contribute to distinct secretory products, whereas the ductal epithelium further modifies saliva composition through ion and fluid transport. However, the heterogeneous distribution and close spatial organization of acinar subtypes can complicate histological interpretation of protein expression patterns, highlighting the need for more detailed mapping of the functional units of the salivary gland.

To address this, a salivary gland antibody panel was designed to distinguish glandular and ductal compartments, including serous glandular cells, mucous glandular cells, small ducts, large ducts, and ionocytes (Figure 3d), with the latter representing specialized epithelial cells involved in ion transport and regulation of fluid composition. Using this panel, 119 proteins have been analyzed in version 25 of the HPA Tissue Resource (Table S1). Mapping candidate protein expression to defined glandular and ductal contexts can aid understanding of salivary epithelial specialization and support studies of proteins involved in secretion, ion transport, mucosal defense, and salivary gland function.

Examples of proteins analyzed using the salivary gland panel include SLC26A9 and TFF3. SLC26A9 (FE1) is a chloride channel and transporter previously described in epithelial tissues, including airway and intestinal epithelia, where it has been reported to interact functionally with CFTR (Wang et al., 2023). Previous studies have identified SLC26A9 expression in salivary gland ducts, consistent with a role in epithelial ion transport. Using the salivary gland panel, we confirm localization of SLC26A9 to the luminal membrane of large ducts, supporting its association with ductal ion and fluid regulation in human salivary gland (Figure 3e). TFF3 (FE4) belongs to the trefoil factor family, a group of secreted peptides involved in protection and repair of mucosal surfaces (Hoffmann, 2021). In salivary gland, TFF3 has been reported as the predominant trefoil factor peptide and may occur both as a homodimer and in complex with FCGBP, although its precise role in salivary gland function remains incompletely understood. Our mIHC analysis localizes TFF3 to mucinous acini, supporting its association with mucosal protection (Figure 3f).

## 6 ENDOCRINE CELL POPULATIONS IN PANCREAS

The pancreas combines digestive and endocrine functions, with the islets of Langerhans forming specialized but relatively scarce compartments embedded within the much larger exocrine pancreas. Despite representing only a small fraction of the total pancreatic tissue, the islets are essential for hormone secretion and metabolic regulation. Within the islets, closely associated endocrine cell types coordinate glucose homeostasis through the release of distinct hormones, including insulin from beta cells, glucagon from alpha cells, somatostatin from delta cells, pancreatic polypeptide from PP cells, and ghrelin from epsilon cells. Because these cell populations are both rare in the tissue as a whole and intermingled within individual islets, they cannot be reliably distinguished by histology alone. Multiplex profiling is therefore particularly important for assigning protein expression to specific islet cell types, especially for proteins with unknown or poorly characterized expression patterns.

To resolve endocrine protein expression in the HPA multiplex tissue resource, a pancreas antibody panel was designed to distinguish the main islet cell populations and their organization within the islet microenvironment (Table 1 and Figure 3g). Using this panel, 77 proteins have been analyzed in version 25 of the HPA Tissue Resource (Table S1). By assigning candidate protein expression to defined endocrine cell types, the dataset supports studies of islet cell specialization, hormone regulation, and pancreatic endocrine function, with relevance for understanding metabolic disease mechanisms such as diabetes.

Examples of proteins analyzed using the pancreas panel include IAPP and ZKSCAN8. IAPP (FE2), also known as islet amyloid polypeptide, is a well‐characterized peptide hormone co‐secreted with insulin from pancreatic beta cells and has been implicated in islet amyloid formation in type 2 diabetes (Atkinson et al., 2020). In agreement with its known biology, our mIHC analysis shows IAPP localization in beta cells (Figure 3h). ZKSCAN8 (FE3) is a zinc finger transcription factor with limited functional characterization and currently sparse literature support in relation to pancreatic biology (Zhou et al., 2023). Using mIHC, ZKSCAN8 showed selective localization to alpha cells, indicating a restricted expression pattern within the endocrine pancreas (Figure 3i).

## 7 FUNCTIONAL REGIONS OF THE NEPHRON IN KIDNEY

Kidney function relies on a highly ordered architecture in which filtration, reabsorption, secretion, and urine concentration are carried out by specialized compartments of the nephron and associated vasculature. The glomerulus forms the filtration unit, whereas the tubular system contains functionally distinct segments responsible for selective transport of water, ions, metabolites, and waste products. Many of these compartments are closely positioned within the tissue, and several tubular and glomerular cell types can be difficult to distinguish by morphology alone.

The kidney antibody panel in the HPA multiplex tissue resource was developed to resolve protein expression across major renal structures, including podocytes, endothelial cells, proximal tubules, distal tubules, and collecting ducts (Table 1 and Figure 3j). In version 25 of the HPA Tissue Resource, the kidney antibody panel has been used to analyze 162 different candidate proteins (Table S1), supporting studies of segment‐specific transport, filtration biology, epithelial specialization, and kidney‐related disease mechanisms.

One of the analyzed proteins is NBC1, encoded by the gene SLC4A4 (FE2), which is a key regulator of bicarbonate secretion and absorption (Burnham et al., 1997). Mutations in the gene cause proximal renal tubular acidosis (Palmer et al., 2021). Here, we confirm protein localization exclusively in the proximal tubules (Figure 3k). Another examined protein is DYNC1I1 (FE1), an intermediate chain of the dynein 1 complex, which facilitates transport along microtubules (Rao & Gennerich, 2024). According to mIHC, DYNC1I1 is localized to podocytes in glomeruli, which is corroborated by scRNA‐seq data (Figure 3l).

## 8 NAVIGATING THE MULTIPLEX TISSUE DATA IN THE HUMAN PROTEIN ATLAS

**FIGURE 4:**
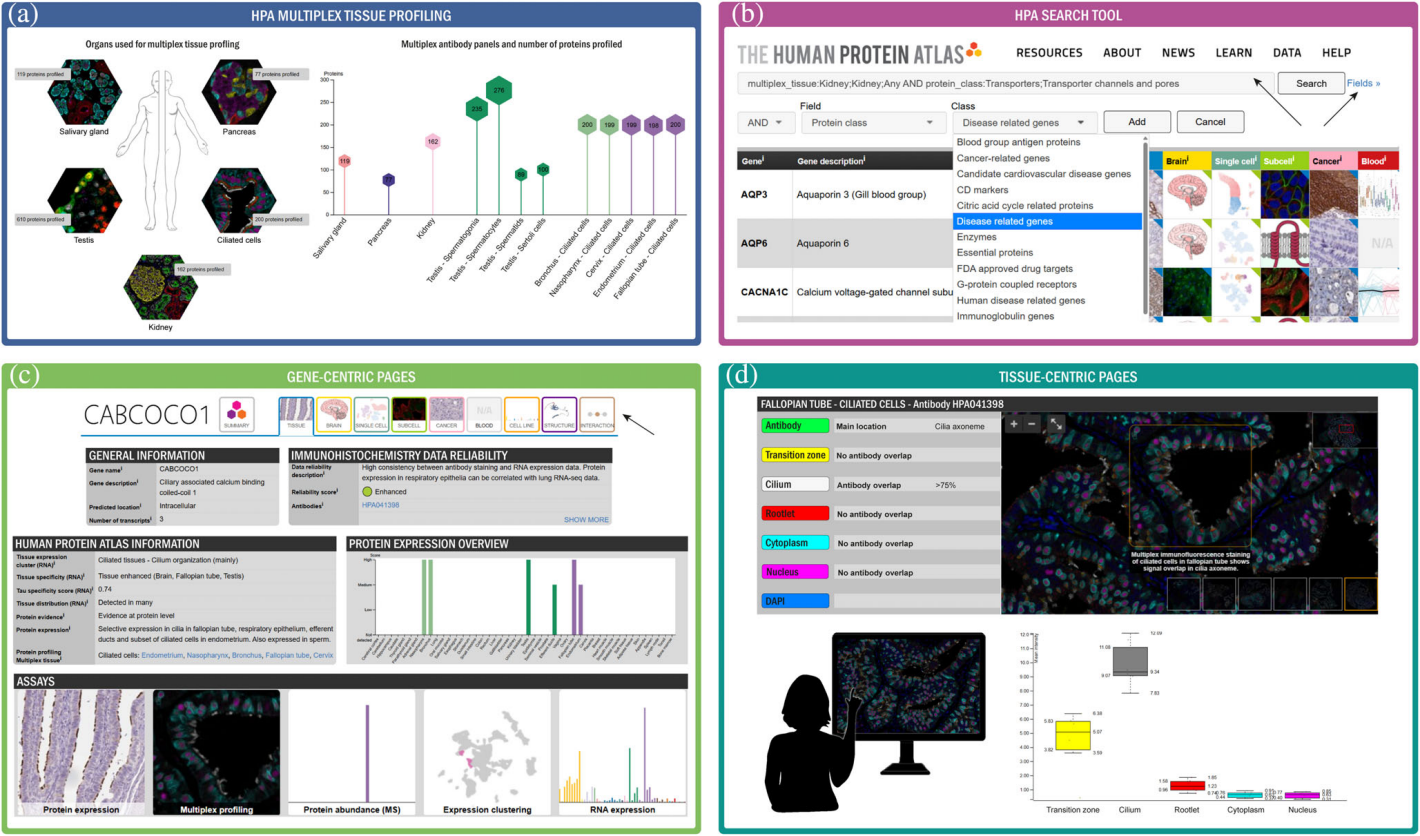
Access and interpretation of multiplex tissue profiling data in the HPA. (a) Overview of the main HPA multiplex tissue resource landing pages, showing an overview of the organs and tissue systems currently included, as well as the number of proteins profiled within each multiplex antibody panel. (b) Proteins analyzed by multiplex tissue profiling can be identified through the HPA search tool and combined with additional search criteria using free text or certain fields (see arrows), such as protein class, tissue context, or disease‐related annotations. (c) Multiplex tissue profiling data are integrated into gene‐centric pages together with other assays including conventional IHC, MS‐based protein abundance and RNA expression, enabling candidate protein localization to be evaluated in the context of complementary transcriptomic and antibody‐based evidence. Links to other HPA resources (see arrow) aid in further functional interpretation. (d) Tissue‐centric pages provide access to interactive high‐resolution images with the possibility to toggle on and off different fluorescent channels, as well as manual annotation of candidate protein localization and automated image analysis results. Candidate protein expression is interpreted in relation to overlap with antibody panel markers, allowing assignment to defined tissue, cellular, or subcellular compartments.

The multiplex tissue profiling data is publicly available in the HPA database, www.proteinatlas.org, where it is integrated with the broader Tissue Resource and connected to complementary transcriptomic and antibody‐based datasets (Figure 4). The data can be explored both from a gene‐centric perspective, where available information for a specific protein is collected on the corresponding gene page, and from a tissue or panel‐centric perspective, where proteins analyzed within a specific multiplex antibody panel can be browsed together.

Lists of proteins analyzed by mIHC can be accessed from the dedicated multiplex tissue profiling landing pages within the Tissue Resource (Figure 4a). These pages provide an overview of the available antibody panels and the tissues or structures analyzed. From these pages, users can learn about the underlying datasets, examine selected protein examples and navigate to panel‐specific protein lists to explore corresponding multiplex images and annotations. Proteins included in the multiplex tissue resource can also be identified through the HPA search engine (Figure 4b), allowing users to combine mIHC information with additional search criteria, such as, for example, tissue specificity, transcript abundance, protein class, or protein interactions, to identify proteins of interest within specific biological or technical contexts.

The multiplex tissue profiling data is also accessible from individual gene pages (Figure 4c). For genes included in the resource, each gene page in the Tissue Resource provides access to the corresponding mIHC results alongside conventional IHC data and RNA expression data. This allows users to compare mIHC‐based localization with other available information within the same gene‐centric framework. From the gene‐centric page, the primary data and all high‐resolution images can be further accessed in tissue‐centric pages (Figure 4d).

A central feature of the multiplex tissue resource is the protein expression in relation to the antibody panel markers. Candidate protein staining is manually interpreted through overlap and co‐localization with panel markers defining specific cell types, cell states, tissue compartments, or subcellular structures (Figure 4d). The resulting annotations include the main location, representing the primary cell type, cell state, or compartment in which the protein is detected, and additional locations, when staining is observed in more than one relevant cellular context but to a lesser extent. The overlap information indicates how the candidate protein relates spatially to established panel markers and provides the basis for assigning protein expression to defined biological structures.

In addition to manual annotation, quantitative automated image analysis data is available for selected panels on the tissue‐centric pages. For the ciliated cell panel, proteins can be evaluated in relation to defined ciliary compartments (Figure 4d). Similar analyses have been performed for the spermatogonia, spermatocyte, and spermatid panels, enabling assessment of protein expression across defined stages throughout spermatogenesis. Computational image analysis was performed on 40× magnification TIFF image stacks, with one image generated for each multiplex channel and each TMA core analyzed separately. The images were first subjected to compartment or cell segmentation, followed by downstream analysis of multiplex signal overlap. Signal overlap was analyzed using ImageJ Fiji for the cilia panel and Ilastik for the spermatogonia, spermatocyte, and spermatid panels. These results complement the manual annotations of main and additional locations and are presented alongside the images, allowing users to compare visual interpretation with quantitative summaries at the same biological resolution.

The multiplex tissue profiling pages are further connected to other parts of the HPA through the gene‐centric pages. From each gene page, users can navigate via tabs, buttons, and links to complementary information, including single‐cell level data, subcellular localization, cancer‐related data, blood profiling in health and disease, and extended information on protein structure and interactions (Figure 4c). This integrated navigation is important because protein function cannot be fully understood from a single data type alone. By placing spatial protein expression in the context of other molecular, cellular, and disease‐related information, the HPA enables users to move from descriptive localization toward broader biological interpretation. In this way, the multiplex tissue profiling data contributes not only to cell type and cell state‐specific protein annotation, but also to the generation of functional hypotheses about how proteins contribute to normal tissue organization, specialized cellular processes, and disease mechanisms.

## 9 DISCUSSION

The multiplex tissue resource in the HPA represents an extension of classical IHC‐based tissue profiling into a higher resolution spatial proteomics framework. By combining validated antibodies, mIHC, tissue‐level expertise, and computational image analysis, the resource enables proteins to be interpreted within defined cellular, subcellular, and tissue‐specific contexts. Because protein function is closely linked to cellular context and subcellular localization, spatially resolved information is essential for understanding how proteins contribute to tissue organization and specialized cellular processes. The HPA multiplex tissue resource therefore provides a tool for moving from protein detection toward spatially resolved protein annotation in intact human tissues.

A major strength of the resource is that it builds directly on the large‐scale antibody validation and classical IHC efforts previously established within the HPA Tissue Resource. The availability of optimized antibodies, prior chromogenic IHC data, a standardized tissue profiling pipeline, and expert‐curated tissue annotations provides a strong foundation for multiplex panel development. This prior knowledge is essential because mIHC is technically more demanding than single‐marker IHC: antibodies must retain specificity under multiplex conditions, tolerate repeated staining and stripping cycles, and be combined in a way that preserves both signal quality and biological interpretability. Thus, the multiplex tissue resource is not a replacement for classical IHC, but rather a natural progression of the HPA strategy, in which single‐marker atlas‐scale profiling serves as the foundation for more detailed spatial and quantitative protein characterization.

The integration of scRNA‐seq and prior biological knowledge into antibody panel design is another key feature of the resource. Single‐cell transcriptomics provides an important framework for defining cell types and cell states, particularly in tissues with continuous differentiation processes or rare cell populations. However, transcriptomic data alone cannot determine whether a candidate marker is translated into protein, where the protein is localized, or whether it is present in the expected cellular context. mIHC addresses these limitations by enabling direct visualization of protein expression in intact tissue sections. In this way, the resource enables cross‐validation between transcriptomics and proteomics: scRNA‐seq guides panel design and candidate selection, while mIHC validates and refines transcriptomics‐based cell type annotations at the protein level.

The examples included in the current resource illustrate how this approach can generate biological insight across diverse tissue systems. In testis, mIHC enables protein expression to be mapped across defined stages of spermatogenesis and Sertoli cell compartments, providing a spatial framework for studying male fertility and germ cell development. In ciliated epithelia, the analysis of multiple tissues with motile ciliated cells enables comparison of both shared and tissue‐specific ciliary protein localization patterns. In salivary gland, pancreas, and kidney, the panels support interpretation of protein expression in relation to functionally specialized compartments. Together, these examples demonstrate how mIHC can help assign proteins to biologically meaningful structures that are difficult to resolve using morphology, conventional IHC, or transcriptomics alone.

The resource is particularly valuable for proteins with limited prior characterization, including those highlighted by the HPP Grand Challenge and FE scores as having limited functional evidence. For such proteins, spatial localization alone is not sufficient to determine function, but it can aid in generating a more focused hypothesis about their potential role, especially for proteins with a restricted localization. Similarly, detection of a protein across multiple cell types, tissue compartments, or subcellular locations may generate hypotheses about secretion, uptake, transport or cell–cell communication. However, because the current mIHC data represent fixed tissue snapshots, they cannot directly demonstrate dynamic processes such as intercellular protein shuttling, which require dedicated functional or time‐resolved experiments. The HPA multiplex tissue resource therefore contributes to functional annotation by placing understudied proteins into spatial and cellular context, guiding more specialized mechanistic studies beyond protein detection alone.

Another important aspect of the resource is the combination of manual interpretation and automated image analysis. Manual annotation remains essential for assessing complex staining patterns, tissue morphology, and biological plausibility. To support reproducibility and reduce observer‐dependent variation, manual annotations were performed using standardized annotation criteria, followed by quality control by a second independent observer. At the same time, automated analysis provides a more quantitative layer that can improve consistency, support comparison between proteins and tissues, and enable systematic assessment of cell state or compartment‐specific expression. The current implementation, including automated analysis for selected germ cell and ciliated cell panels, illustrates the potential of combining expert interpretation with computational analysis. As image analysis methods continue to improve, this component will likely become increasingly important for scaling the resource and extracting quantitative information from large multiplex imaging datasets. The quantitative nature of the image analysis data also allows for mRNA‐to‐protein correlation analyses. Interestingly, a large number of the testicular proteins analyzed with the germ cell proteins showed a disconcordance between mRNA and protein levels at a cell state‐specific level, with peak protein expression observed at a later cell state as compared to mRNA levels (Hikmet et al., 2024). Further efforts on mRNA‐to‐protein concordance should be explored in future follow‐up studies using the HPA multiplex tissue resource data.

The design of the HPA multiplex tissue resource reflects a balance between biological resolution, technical robustness, and scalability. There are several other high‐plex spatial proteomics methods that can measure larger numbers of proteins within a single tissue section, including CODEX/PhenoCycler Fusion (Black et al., 2021), which uses DNA‐barcoded antibodies and cyclic fluorescent readout; multiplexed ion beam imaging (MIBI) (Rao & Gennerich, 2024), which uses metal isotope‐labeled antibodies detected by ion beam mass spectrometry; and imaging mass cytometry (IMC) (Liu et al., 2022), which combines metal‐tagged antibodies with laser ablation and mass cytometry‐based detection. These approaches provide powerful high‐dimensional spatial data, but often require specialized instrumentation, antibody conjugation or barcoding, and extensive panel optimization. In contrast, the mIHC strategy used in the HPA resource builds on existing antibody validation and does not require custom conjugation of each antibody, making it particularly suitable for a scalable atlas framework aimed at analyzing large numbers of candidate proteins across tissues using robust and interpretable panels.

At the same time, the scalability of this approach depends on careful management of several technical and biological considerations. The number of markers that can be included in each panel is lower than in some high‐plex platforms, making panel composition particularly important. Antibody performance may also differ between chromogenic IHC and mIHC conditions, and repeated staining, heating, and stripping cycles can influence epitope accessibility or signal intensity. These factors are addressed through single‐plex validation, optimization of antibody dilution, fluorophore assignment, and staining order, together with comparison to existing HPA IHC reference patterns. From a biological perspective, the selected panel markers must provide sufficient separation between relevant cell types, cell states, or subcellular compartments, since proteins with broad or highly overlapping expression patterns may be more difficult to interpret. When a single panel is not sufficient to resolve the full biological complexity of a tissue, multiple complementary panels can be designed for the same organ, as illustrated by the four testis panels targeting different stages of spermatogenesis and Sertoli cell compartments. This modular strategy allows biological complexity to be addressed stepwise while maintaining a workflow that remains scalable across many candidate proteins and tissues.

A further strength of the HPA is that multiplex tissue profiling is embedded within the broader public database, allowing spatial protein expression to be interpreted together with complementary molecular, tissue‐level and single cell‐level information. Classical IHC and mIHC provide different but complementary levels of resolution: classical IHC offers a broad body‐wide overview of protein expression across many tissues and major cell types, whereas multiplex tissue profiling enables deeper investigation of selected structures by relating candidate proteins to defined panel markers. Existing HPA bulk and scRNA‐seq data add information on tissue and cell type‐specific RNA expression, while the deep visual proteomics (DVP) (Weiss et al., 2026) data recently added to HPA version 25.1 further expands this framework by linking image‐defined cell phenotypes to proteome‐wide molecular profiles across human tissues and cell types. The public availability of these interconnected data layers enables users to move beyond isolated localization patterns toward a broader understanding of protein expression, localization, and potential function in human tissues.

Future development of the resource could further increase its value for protein science. Expansion to additional organ systems, tissues, disease states, developmental stages, and physiological conditions would broaden the biological scope of the atlas and enable more systematic comparisons between normal and altered tissue organization. Future panels could also strengthen biological interpretation by including markers of cellular activity, such as signaling pathway activation, metabolic specialization, or communication between neighboring cells. In addition, spatial transcriptomics, although not currently part of the HPA multiplex tissue resource, represents a complementary approach that has the potential to further connect targeted protein localization with broader spatial gene expression programs in future studies. For example, the use of spatial transcriptomics to guide marker selection could improve detection of rare cell populations and decipher more precisely the location of different cell types by providing an anatomical context not available with scRNA‐seq alone. There is significant methodological development in this area facilitating ever‐higher resolutions and future prospect of possibly analyzing the same sample with a combination of several complementary techniques. Together with continued advances in image analysis and multimodal data integration, these developments would further enhance the ability of the HPA to support functional interpretation of protein expression in tissue context.

In summary, the HPA multiplex tissue resource provides a scalable and biologically interpretable approach for spatially resolved protein annotation in human tissues. By building on the antibody foundation of the classical HPA Tissue Resource and combining it with multiplex imaging, transcriptomic guidance, and computational image analysis, the resource enables a high‐resolution cell type, cell state, and compartment‐specific interpretation of protein expression. As the resource expands, it has the potential to become an increasingly important reference for functional protein annotation and a valuable tool for the broader protein science community.

## AUTHOR CONTRIBUTIONS

**Borbala Katona:** Investigation; validation; writing – original draft; formal analysis; data curation. **Rutger Schutten:** Investigation; writing – original draft; formal analysis; methodology. **Filippa Bertilsson:** Investigation; writing – review and editing; visualization; software; data curation; validation. **Feria Hikmet:** Writing – review and editing; software; data curation; methodology; conceptualization; validation. **Per Adelsköld:** Writing – review and editing; visualization; software. **Mattias Forsberg:** Visualization; software; writing – review and editing. **Kalle von Feilitzen:** Visualization; writing – review and editing; formal analysis. **Loren Méar:** Software; supervision; data curation; investigation; writing – review and editing; methodology. **Mathias Uhlén:** Resources; conceptualization; funding acquisition; writing – review and editing. **Cecilia Lindskog:** Writing – original draft; funding acquisition; resources; project administration; supervision; data curation; validation; visualization; methodology; conceptualization.

## CONFLICT OF INTEREST STATEMENT

The authors declare no conflicts of interest.

## Supporting information


**TABLE S1:** List of proteins analyzed with multiplex immunohistochemistry for each of the different antibody panels.

## Data Availability

The data that support the findings of this study are openly available in Human Protein Atlas at https://www.proteinatlas.org.
